# Increased risk of myocarditis and pericarditis and reduced likelihood of severe clinical outcomes associated with COVID-19 vaccination: a cohort study in Lombardy, Italy

**DOI:** 10.1186/s12879-022-07823-3

**Published:** 2022-11-12

**Authors:** Giovanni Corrao, Matteo Franchi, Danilo Cereda, Francesco Bortolan, Olivia Leoni, Eugenio Vignati, Giovanni Pavesi, Andrea Gori

**Affiliations:** 1 National Centre for Healthcare Research and Pharmacoepidemiology, University of Milano-Bicocca, Milan, Italy; 2 Unit of Biostatistics, Epidemiology and Public Health, Department of Statistics and Quantitative Methods, University of Milano-Bicocca, Via Bicocca degli Arcimboldi, 8, Edificio U7, 20126 Milan, Italy; 3 Directorate General for Health, Lombardy Region, Milan, Italy; 4 Welfare Councillor Staff, Lombardy Region, 20124 Milan, Italy; 5 Infectious Diseases Unit, Fondazione Istituto di Ricovero e Cura a Carattere Scientifico (IRCCS) Ca’ Granda Ospedale Maggiore Policlinico, Milan, Italy; 6 Department of Pathophysiology and Transplantation, University of Milan, Milan, Italy; 7 Centre for Multidisciplinary Research in Health Science (MACH), University of Milan, Milan, Italy

**Keywords:** SARS-CoV-2, COVID-19, COVID-19 vaccination, Myocarditis, Pericarditis

## Abstract

**Introduction:**

We aimed to assess harms (post-vaccine myocarditis and pericarditis) and benefits (preventing severe disease) of COVID-19 vaccination.

**Methods:**

We conducted a population-based retrospective cohort study. Using the integrated platform of the vaccination campaign of Lombardy Region (Italy), after the exclusion of 24,188 individuals not beneficiaries of the Regional Health Service, 9,184,146 citizens candidates to vaccine at December 27, 2020 were followed until November 30, 2021 (the loss to follow-up rate was 0.5%). From the date of administration of each vaccine dose to day 28 post-administration, three periods that covered exposure to the first, second, and third dose were defined. The benefit–risk profile of vaccines was performed by comparing the number needed to harm (NNH) and number needed to treat (NNT) by sex, age, and vaccine type.

**Results:**

Incidence rates of myocarditis were 9.9 and 5.2 per million person-months during the exposure and no-exposure periods, respectively, and the incidence rates of pericarditis were 19.5 and 15.9 per million person-months, respectively. The risk of myocarditis was highest following exposure to the second dose of the Moderna vaccine (adjusted HR: 5.5, 95% CI: 3.7 to 8.1). Exposure to the Moderna vaccine was also associated with an increased risk of pericarditis (adjusted HR 2.2, 1.5 to 3.1). NNT was higher than NNH (9471 vs. 7213) for 16 to 19-year-old men who received the Moderna vaccine, while all other sex, age, and vaccine subgroups had a favourable harm-benefit profile.

**Conclusions:**

Men 16 to 19 years of age has the highest rates of myocarditis within a few days after receiving the Moderna vaccines. The balance between harms and benefits was almost always in favour of vaccination.

**Supplementary Information:**

The online version contains supplementary material available at 10.1186/s12879-022-07823-3.

## Introduction

The scientific community and the pharmaceutical industry, backed by government support, have devoted massive efforts to the development of efficacious and safe vaccines against severe acute respiratory syndrome coronavirus 2 (SARS-CoV-2) [1]. Four vaccines are currently approved for use against coronavirus disease 2019 (COVID-19) in the European Union: two mRNA-based vaccines (manufactured by Pfizer-BioNTech and Moderna) and two based on an adenovirus vector (manufactured by Oxford-AstraZeneca and Janssen) [2]. Phase 3 clinical trials showed that all four COVID-19 vaccines are efficacious and have an acceptable safety profile [3–6]. However, given the inherent limitations of clinical trials to assess vaccine safety as a result of healthier-than-average participants and low power to identify less common adverse events, post-marketing surveillance and real-life study are required to monitor vaccine safety in real-world settings [7]. This issue was of particular concern at the beginning of the vaccination campaign, when pharmacovigilance studies suggested that adenovirus-vectored vaccines could have been responsible of rare thromboembolic events [8, 9]. However, a population-based cohort study conducted in the same setting as compared to the current study, aimed to evaluate the balance between benefits and harms of citizens vaccinated with available adenovirus-vectored vaccines, found a favourable risk benefit profile [10].

Beginning with initial reports of myocarditis following mRNA vaccination [11], additional pharmacovigilance, health system surveillance, and case series studies have suggested an association between SARS-CoV-2 vaccination and development of both myocarditis and pericarditis [7, 12–25]. However, formal epidemiologic studies comparing observed and expected cases of myocarditis and other clinical outcomes like pericarditis, are sparse and inconsistent [26–28].

While myocarditis following COVID-19 vaccination is rare, it more frequently affects younger people. Since more severe disease rarely impacts this group, the vaccine is expected to provide more protection against serious disease in older than young people. This raises concern about the balance between vaccine benefits and harms. It should be emphasized that because the vaccine’s ability to prevent COVID-19 disease is expected to increase as SARS-CoV-2 spreads, the benefit-harm balance is likely to change over time. To the best of our knowledge, this issue, while critically important, has not yet been investigated.

This population-based retrospective cohort study used the platform specifically designed to monitor and assess vaccination in the Italian Lombardy Region [29] to compare harms associated with vaccine exposure, including myocarditis and pericarditis, with the benefits of vaccination, including the prevention of severe disease in particular subpopulations. Aside from estimating the relationship between vaccine exposure and the short-term risk of myocarditis and pericarditis, a detailed benefit–risk assessment was performed by comparing the Number Needed to Treat (NNT) with the Number Needed to Harm (NNH) under different conditions.

## Methods

### The Lombardy Vaccine Integrated Platform

Four data sources were used to develop the Lombardy Vaccine Integrated Platform. First, a vaccine registry was established by the Regional Health Authority beginning on December 27, 2020, to collect individual data on the date, type, and dose of the dispensed vaccine (the vaccine is administered for free to all candidate to vaccination). Second, a registry of patients with a confirmed diagnosis of SARS-CoV-2 infection was established on February 21, 2020 (the date of the first confirmed diagnosis) to monitor SARS-CoV-2 infections, hospital admissions, emergency room visits, and deaths due to COVID-19. Third, a healthcare utilization database that was established in 2000 to collect a variety of information, including inpatient diagnoses supplied by public or private hospitals, and outpatient drug and services supplied by RHS departments, was used to obtain data on the health profile of the study population. Finally, the health registry reports and updates data on patients' status of the Lombardy Regional Health Service (RHS), which includes almost all Lombardy residents, including date and cause of entry (birth, immigration) or exit (death, emigration) from the database. These databases use a unique individual identification code that allows to link each database with each other. To maintain privacy, each identification code is anonymized so that individuals can only be identified by the Regional Health Authority upon request from judicial authorities.

### Cohort selection and covariates

Vaccine candidates (aged 12 years or older) who were not beneficiaries of the RHS or who became beneficiaries after January 1, 2019 (N = 24,188), were excluded from the study. The remaining 9,184,146 individuals were included in the study cohort and the date and type of vaccine administered from December 27, 2020 (index date) to November 30, 2021 (observation period) were recorded for each person. Sex, age, and clinical profile were recorded at the index date. Hospital admissions and drug prescriptions obtained within 2 years before the index date were used to investigate nine specific cardiovascular diseases. A complete list of these diseases, along with their accompanying ICD-9 and ATC codes, in addition to other conditions and exposures considered during this study, is provided in Additional file 1: Table S1.

### Measuring vaccine harms

The time of observation for each cohort member was partitioned into four exposure categories to assess the risk of vaccine harm. From the date of administration of each vaccine dose to day 28 post-administration, three periods that covered exposure to the first, second, and third dose were defined. The periods that remained uncovered by vaccine exposure were defined as no exposure to the risk of vaccine harm. Hospital admissions associated with a diagnosis of myocarditis (ICD9-CM codes 422.xx and 429.0) or pericarditis (ICD9-CM codes 420.xx) that occurred during the observation period were identified and denoted as the vaccine harm outcome of interest.

Cohort members accumulated person-months of observation from December 27, 2020 until outcome onset, death, emigration out of the region, or November 30, 2021, whichever came first. The incidence rates of myocarditis and pericarditis were separately calculated as the ratio between the number of outcomes and the number of person-months occurred during the period of interest (exposure or no exposure to the risk of vaccine harm). Where relevant, rates were stratified by sex, age category, and vaccine dose and type.

Cox proportional hazard models were fitted for estimating the hazard ratio (HR) and 95% confidence interval (CI) of the defined outcomes, associated with exposure to the first, second, and third dose of each vaccine type, compared to no exposure. As vaccine exposure changed over time, assessment of its effect required consideration of this change. This was performed by fitting the Cox model with dummy factors for exposure categories that were expressed as time-dependent covariates. Adjustments were made for sex, age, and cardiovascular comorbidities.

### Measuring vaccine benefits

The time of observation of each cohort member was partitioned into two categories of exposure to assess vaccine benefit. The first category included the period 14 days after the first vaccine dose until November 30, 2021, and was based on the assumption that clinically significant immunity is achieved 2 weeks after receiving the COVID-19 vaccine [30]. The second category included time-periods uncovered by vaccine exposure and was defined as no exposure to vaccine protection.

A composite outcome that included hospital admission, including patients in intensive care units, and deaths attributed to COVID-19, whichever occurred first, was tracked for each individual and denoted as the outcome of interest. Cohort members accumulated person-months of observation from December 27, 2020, until outcome onset, death, out of region emigration, or November 30, 2021, whichever came first. The incidence rates of severe COVID-19 disease were calculated as the ratio between the number of outcomes and the number of person-months occurred during the period of interest (exposure or no exposure to vaccine). Incidence rates were stratified by sex, age category, and vaccine type.

### Balancing harms and benefits

Different metrics were used to compare the harms and benefits of vaccine exposure. In particular, the NNH was considered the number of individuals who would need to be vaccinated to generate a case of myocarditis, while the NNT was considered the number of individuals who should be vaccinated to avoid a case of severe COVID-19 disease [10]. Therefore, a desirable vaccination profile was defined as a low NNT and a high NNH [31]. NNT was calculated as the reciprocal of the absolute difference in incidence rates of myocarditis during the exposure and no exposure periods. NNH was calculated as the reciprocal of the absolute difference in incidence rates of severe COVID-19 diseases during the no exposure and the exposure periods. The corresponding 95% CI were calculated as the inverse of the CI around the absolute incidence rate difference [32]. As a secondary analysis, NNH and NNT were re-calculated by excluding cohort members who did not receive the vaccine during the entire observation period.

## Results

### Study cohort

**Table ****1** shows the selected baseline characteristics of the 9,184,146 vaccine recipients on December 27, 2020, and their vaccination status on November 30, 2021 (41,463 (0.5%) cohort members were lost to follow-up because they emigrated out of the Region). Men were 51.3% of the overall population. The percentage of individuals aged 12–15, 16–19, 20–29 and ≥ 30 were, respectively, 4.4%, 4.2%, 10.7% and 80.7%. Almost one in four recipients had a cardiac comorbidity. Globally, more than 16 million doses were administered over the 11-month period since the first vaccine was given. About 90% and 80% of recipients received one and two vaccine doses, respectively. More than half of individuals received the Pfizer-BioNTech vaccine, 12.5% received Oxford-AstraZeneca, 10% received Moderna, and even fewer individuals received Jansen. Just over 10% of recipients received the booster dose.

**Table 1:** Baseline characters of the 9,184,146 vaccine recipients on December 27, 2020 and vaccination status on November 30, 2021

Baseline characteristics	
Sex	
Men	4,715,243 (51.3%)
Women	4,468,903 (48.7%)
Age categories	
12–15 years	401,186 (4.4%)
16–19 years	387,685 (4.2%)
20–29 years	977,758 (10.7%)
30–39 years	1,130,699 (12.3%)
40–49 years	1,493,328 (16.3%)
50–59 years	1,665,591 (18.1%)
60–69 years	1,254,101 (13.7%)
70–79 years	1,026,382 (11.2%)
80 + years	847,416 (9.2%)
Cardiac comorbidities	
Arrhythmic myocardiopathy	237,941 (2.6%)
Arterial vascular disease	76,321 (0.8%)
Familial and non-familial hypercholesterolaemia	274,477 (3.0%)
Heart failure	161,551 (1.8%)
Hypertension	1,285,967 (14.0%)
Ischaemic heart disease	246,631 (2.7%)
Non-arrhythmic myocardiopathy	245,683 (2.7%)
Valvular heart disease	69,656 (0.8%)
Venous vascular disease	41,184 (0.5%)
At least one cardiac comorbidity	2,255,136 (24.5%)

Vaccine status	
First dose	7,907,765 (86.1%)
Oxford-AstraZeneca	1,223,681 (13.3%)
Janssen	289,989 (3.2%)
Moderna	1,024,935 (11.2%)
Pfizer-BioNTech	5,369,160 (58.5%)
Second dose	7,165,379 (78.0%)
Oxford-AstraZeneca	1,041,884 (11.3%)
Moderna	970,629 (10.6%)
Pfizer-BioNTech	5,152,866 (56.1%)
Third dose (booster)	1,052,469 (11.5%)
Moderna	108,319 (1.2%)
Pfizer-BioNTech	944,150 (10.3%)

### Harm-related outcomes

Of the cohort members, 587 and 1658 experienced myocarditis and pericarditis during the observation period, respectively. Of these patients, 12 and 28 died during their hospital stay, respectively. No individuals who experienced events during person-months of exposure to the risk of vaccine harm (i.e. 28 days after vaccine administration) died during hospitalization. The incidence rates of myocarditis and pericarditis were 5.8 and 16.2 per million person-months, respectively. The incidence of myocarditis was almost double during exposure to the risk of vaccine harm than no exposure, with a corresponding number of cases and incidence rate of 124 and 9.9 every million person-months, respectively, for exposed individuals and 463 and 5.2 per million person-months, respectively, for those with no exposure. The incidence of pericarditis was 1.2-fold higher during exposure than no exposure, with the corresponding number of cases and incidence rate of 245 and 19.5 per million person-months, respectively, for exposed individuals, and 1413 and 15.9 per million person-months, respectively, for those with no exposure.

**Fig. 1:**
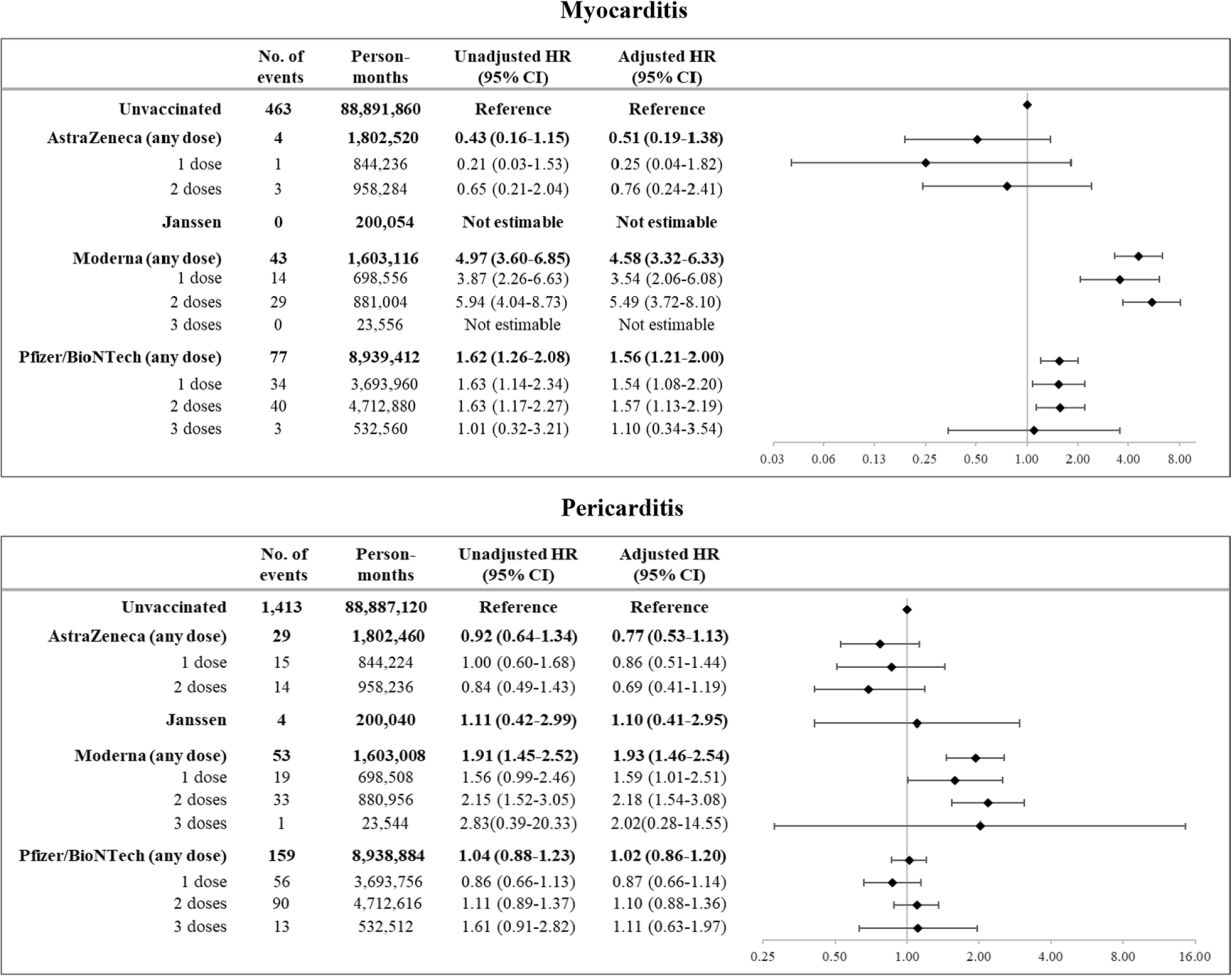
Forest plot showing the association between exposure to the first, second, and third dose of vaccines manufactured by Oxford-AstraZeneca, Jansen, Moderna, and Pfizer-BioNTech and the risk of myocarditis (top panel) and pericarditis (bottom panel). Cox proportional hazard models were used for separately estimating the Hazard Ratio (HR) of myocarditis and pericarditis, together with the 95% confidence interval (CI), associated with time-dependent exposure to the vaccine. The model was adjusted for available demographic and clinical characteristics

Exposure to an adenovirus vector-based vaccine was not associated with an increased risk of myocarditis (**Fig. ****1**, top panel). In contrast, exposure to the first and second doses of mRNA-based vaccines was associated with an increased risk of myocarditis, in particular after receipt of the second dose of Moderna (adjusted HR 5.5, 95% CI 3.7 to 8.1). There was no evidence that exposure to an adenovirus vector-based vaccine or the Pfizer-BioNTech mRNA vaccine was associated with an increased risk of pericarditis (**Fig. ****1**, bottom panel). In contrast, exposure to the first and particularly the second dose of Moderna was associated with an increased risk of pericarditis, although with lower strength than that observed for myocarditis (adjusted HR 2.2, 95% CI 1.5 to 3.1).

**Fig. 2:**
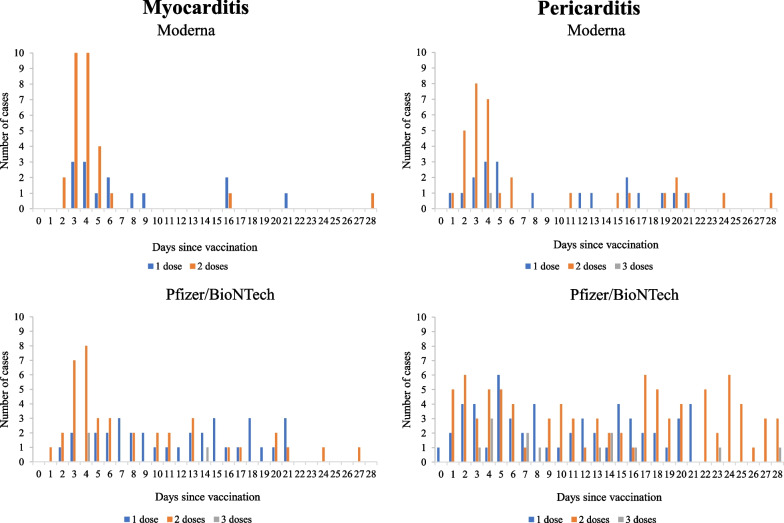
Timing of myocarditis and pericarditis hospitalization after receiving the first, second, and third dose of the Moderna and Pfizer-BioNTech vaccine

The timing of myocarditis and pericarditis presentation after vaccine administration is shown in **Fig. ****2**. Myocarditis primarily occurred within a few days after receipt of the second dose of either Moderna or Pfizer-BioNTech (27 of 29 cases and 24 of 40 cases one week after receipt of the Moderna and Pfizer-BioNTech vaccine, respectively). Pericarditis also tended to occur within a few days after the second dose of Moderna (24 of 33 cases one week after vaccine receipt). In contrast, pericarditis that occurred following receipt of the Pfizer-BioNTech vaccine did not follow a temporal trend.

### Balancing harms and benefits

The highest rate of myocarditis occurred among men 16 to 19 years of age following exposure to the Moderna vaccine (Table 2). This group had a 9.5-fold higher incidence than men of the same age during time periods with no vaccine exposure. It should be emphasized that this subpopulation was the only one to have a higher NNT than NNH (9471 vs. 7213, respectively). All other subpopulations defined by sex, age category and vaccine type assessed in this study had a favourable harm-benefit profile with an NNH and NNT ratio ranging from 1.5 (men 12–15 years of age receiving Pfizer-BioNTech) to 962.5 (men > 30 years of age receiving Pfizer-BioNTech). These findings were confirmed after excluding 1,276,381 individuals from the study cohort who did not receive a vaccine during the entire observation period (Table 3).

**Table 2:** Harm-benefit profile of the Moderna and Pfizer-BioNTech vaccines by sex and age category

Men receiving the Moderna vaccine												
Harms						Age categories	Benefits					
No exposure^a^		Exposure^a^		Rate difference^c^	Number needed to harm (95% CI)^e^		Number needed to treat (95% CI)^e^	Rate difference	Exposure^b^		No exposure^b^	
# cases^c^	Incidence rate^c^	# cases^c^	Incidence rate^c^						Incidence rate^d^	# cases^d^	Incidence rate^d^	# cases^d^
12	5.8	1	33.1	27.3	36,680 (10,854 to − 26,596)	12–15 years	20,008 (9980 to − 3,529,828)	50.0	35.0	2	84.9	155
32	16.3	6	154.9	138.6	7213 (3807 to 68,776)	16–19 years	9471 (7513 to 12,852)	105.6	11.2	1	116.8	182
70	14.2	14	94.8	80.6	12,414 (7675 to − 32,468)	20–29 years	6764 (5790 to 8130)	147.9	41.8	16	189.7	693
203	5.9	14	23.7	17.8	56,106 (33,025 to 186,428)	+ 30 years	911 (890 to 933)	1097.7	206.9	389	1304.6	27,939

Women receiving the Moderna vaccine												
Harms						Age categories	Benefits					
No exposure^a^		Exposure^a^		Rate difference^c^	Number needed to harm (95% CI)^e^		Number needed to treat (95% CI)^e^	Rate difference	Exposure^b^		No exposure^b^	
# cases^c^	Incidence rate^c^	# cases^c^	Incidence rate^c^						Incidence rate^d^	# cases^d^	Incidence rate^d^	# cases^d^
3	1.5	0	0.0	–	–	12–15 years	27,522 (11,049 to − 56,054)	36.3	38.0	2	74.3	127
8	4.4	1	29.9	25.5	39,184 (11,882 to − 30,193)	16–19 years	7285 (5907 to 9506)	137.3	12.8	1	150.0	216
18	3.9	3	25.2	21.3	46,920 (20,044 to − 137,646)	20–29 years	4279 (3809 to 4980)	233.7	43.9	14	277.6	908
117	3.1	4	6.5	3.3	298,908 (102,754 to − 328,839)	+ 30 years	1,265 (1228 to 1305)	790.2	216.9	454	1007.1	22,328

Men receiving the Pfizer-BioNTech vaccine												
Harms						Age categories	Benefits					
No exposure^a^		Exposure^a^		Rate difference^c^	Number needed to harm (95% CI)^e^		Number needed to treat (95% CI)^e^	Rate difference	Exposure^b^		No exposure^b^	
# cases^c^	Incidence rate^c^	# cases^c^	Incidence rate^c^						Incidence rate^d^	# cases^d^	Incidence rate^d^	# cases^d^
12	5.8	9	46.4	40.6	24,633 (14,069 to 98,9123)	12–15 years	16,543 (12,395 to 24,863)	60.5	24.5	10	84.9	155
32	16.3	8	36.1	19.7	50,633 (22,046 to − 170,561)	16–19 years	11,741 (9294 to 15,929)	85.2	31.6	18	116.8	182
70	14.2	13	25.4	11.2	89,592 (39,448 to − 330,251)	20–29 years	6,395 (5770 to 7174)	156.4	33.3	49	189.7	693
203	5.9	24	7.0	1.1	890,332 (246,731 to − 553,403)	+ 30 years	925 (910 to 940)	1081.1	223.5	2,556	1304.6	27,939

Women receiving the Pfizer-BioNTech vaccine												
Harms							Benefits					
No exposure^a^		Exposure^a^		Rate difference	Number needed to harm (95% CI)^e^	Age categories	Number needed to treat (95% CI)^e^	Rate difference^c^	Exposure^b^		No exposure^b^	
# cases^c^	Incidence rate^c^	# cases^c^	Incidence rate^c^						Incidence rate^d^	# cases^d^	Incidence rate^d^	# cases^d^
3	1.5	0	0.0	–	–	12–15 years	18,560 (13,691 to 28,810)	53.9	20.4	8	74.3	127
8	4.4	2	9.5	5.1	196,329 (53,937 to − 119,175)	16–19 years	10,413 (8071 to 14,658)	96.0	54.0	30	150.0	216
18	3.9	4	8.3	4.4	227,298 (78,616 to − 254,842)	20–29 years	4254 (3906 to 4668)	235.1	42.5	63	277.6	908
117	3.1	17	4.4	1.3	772,323 (287,274 to − 1,121,831)	+ 30 years	1214 (1192 to 1237)	823.5	183.6	2416	1007.1	22,328

The entire cohort of 9,184,146 vaccine recipients on December 27, 2020 was included in this analysis

^a^The observation time for each cohort member was partitioned into two exposure categories to assess the risk of vaccine harm. Starting from the date of administration of each vaccine dose and continuing for 28 days, the exposure period was identified and defined as exposure to the vaccine. The remaining periods (i.e. those uncovered by vaccine exposure) were defined as no exposure to the risk of vaccine harm

^b^Time of observation for each cohort member was partitioned into two categories of exposure to the benefits of vaccine. The first was from the 14 days after the first vaccine dose and November 30, 2021. The remaining time periods (i.e. those uncovered by vaccine exposure) were defined as no exposure to vaccine protection

^c^The first episode of hospital admission with a diagnosis of myocarditis was defined as an outcome event. The incidence rate of myocarditis was calculated as the ratio between the number of outcomes occurring during the period of interest (i.e. exposure or no exposure to the risk of vaccine harm), and the number of person-months accumulating during that period and expressed as the number of cases every million person-months at risk

^d^The first episode of hospital admission, including patients in intensive care units, or deaths attributed to COVID-19 was defined as an outcome event. The incidence rate of severe COVID-19 disease was calculated as the ratio between the number of outcomes occurring during the period of interest (i.e. exposure or no exposure to vaccine protection) and the number of person-months accumulating during that period), and expressed as the number of cases per million person-months at risk

^e^The upper limit of confidence interval has sometimes negative values. Following Altman [29], a negative value of NNT indicates that the vaccine has a harmful effect, a confidence interval spanning from a positive to a negative value of lower and upper limits, respectively, suggests that the study did not offer statistical evidence that vaccination avoids severe COVID-19 outcomes (i.e. NNT was not significantly different from the null in that sex and age category). Analogously, a negative value of NNH indicates that the vaccine doesn’t have a harmful effect, an interval between lower and upper limits which includes the null, suggests that the study did not offer statistical evidence that the vaccine generates myocarditis (i.e., that NNH was not significantly different from the null in that sex and age category)

**Table 3:** Harm-benefit profile of the Moderna and Pfizer-BioNTech vaccines by sex and age category

Individuals receiving the Moderna vaccine				
Men			Women	
Number Needed to Harm	Number Needed to Treat	Age categories	Number Needed to Harm	Number Needed to Treat
30,252	21,876	12 to 15 years	–	33,621
6747	10,317	16 to 19 years	34,997	7861
11,086	7060	20 to 29 years	41,819	4621
47,910	1085	≥ 30 years	210,530	1527

Individuals receiving the Pfizer-BioNTech vaccine				
Men			Women	
Number Needed to Harm	Number Needed to Treat	Age categories	Number Needed to Harm	Number Needed to Treat
21,557	17,800	12 to 15 years	–	21,148
34,095	13,069	16 to 19 years	122,747	10,317
48,036	6660	20 to 29 years	142,873	7060
239,663	1105	≥ 30 years	370,479	1085

The restricted cohort of 7,907,765 vaccine recipients who experienced at least one vaccine administration were included in this analysis

## Discussion

### Main findings

Using the Vaccine Integrated Platform that covered the entire Lombardy population, a strong association was observed between exposure to mRNA-based vaccines and the risk of both myocarditis and pericarditis compared to periods of no exposure. The risk of myocarditis was particularly high after receipt of the second dose of the Moderna vaccine (adjusted HR 5.5, 95% CI: 3.7 to 8.1), lower after the first dose (adjusted HR 3.5, 95% CI: 2.1 to 6.1), and even less following the first and second doses of the Pfizer-BioNTech vaccine (adjusted HR 1.5, 95% CI: 1.1 to 2.2). Increased risk of pericarditis was noticed after receiving the first and second dose of Moderna (adjusted HR, and 95% CI, 1.6, 1.0 to 2.5, and 2.2, 1.5 to 3.1, respectively). These founding should be compared with risk of myocarditis and pericarditis following post-acute COVID-19, which, in a recent US cohort study, was shown to be 5.38-fold and 1.85-fold, respectively, as compared to controls [33]. Adenovirus vector-based vaccines did not increase the risk of myocarditis or pericarditis, and the Pfizer-BioNTech vaccine did not increase the risk of pericarditis. The study was underpowered to estimate the impact of booster doses on harmful outcomes.

The novel approach used in this study for evaluating the balance between harms and benefits of anti-COVID-19 vaccines was previously used in a similar setting, for evaluating a potential increased risk of thromboembolic events in individuals who received the adenovirus-vectored vaccines [10]. In the current study, an unfavourable balance between harms and benefits for men 16 to 19 years of age who received the Moderna vaccine was highlighted. In this subpopulation, NNT was higher than NNH with corresponding values of 9471 and 7213, respectively, considering the entire study cohort, and 10,317 and 6747, respectively, excluding those who did not receive the vaccine during the entire observation period. It should be emphasized, however, that for this “highest risk” category: (i) the incidence rate of myocarditis was only 1.5 new cases every 10,000 vaccinated individuals; (ii) 7213 citizens (6747 in the secondary analysis) should be vaccinated to generate a case of myocarditis; (iii) although the NNT was slightly higher (from 9471 in the primary analysis to 10,317 in the secondary analysis), suggesting an unfavourable balance between harm and benefit, we cannot disregard that harm and benefit outcomes had substantial difference in clinical profile, resolving the former in few days hospital stay without any fatal outcome, while avoiding severe outcomes, including severe acute respiratory syndrome, that sometimes may result in invasive intubation or death; (iv) the NNT for avoiding a case of SARS-CoV-2 infection was only 215 (data not shown), confirming the potential of a vaccine to prevent infection spread, even when there is a small risk of myocarditis. For all other combinations of sex, age and vaccine type, even lower rates and higher NNH, and a more favourable balance toward vaccine benefits was observed. Taken together, these findings suggest that the balance between harm and benefit is largely skewed towards vaccination, even in men 16 to 19 years of age, and vaccines, in particular those that are mRNA based, remain the primary method for fighting the pandemic. Careful monitoring of the harm-benefit profile, however, is strongly recommended. In fact, due to the expected reduction of viral spread, increasing NNT values are expected in the next few months, suggesting that the investigated balance may become less favourable.

As concern the underlying disease mechanism, myocarditis and pericarditis do not represent novel adverse events of vaccination. Previously, other vaccinations have been associated with an increased risk of myocarditis [34]. These findings suggest that the disease mechanism is specific neither for the newly developed mRNA vaccines, nor for the exposure to the SARS-CoV-2 spike protein. Although rare, clinicians should however be aware of the risk of myocarditis and pericarditis, which should be considered in individuals presenting with chest pain within a week after vaccination, in particular among younger individuals [35].

### Comparison with other studies

Previous studies in Israel, Denmark, England, and United States have also shown higher myocarditis incidence after vaccination with the SARS-CoV-2 mRNA vaccine, indicating that this adverse effect is impacting diverse populations [7, 12, 14, 18, 19, 26–28]. In contrast to a recent study out of Denmark [27], but consistent with others, the current study confirmed (i) a higher risk of myocarditis after Pfizer-BioNTech vaccination, although less than that observed after Moderna, (ii) that the second dose of Moderna was associated with a higher risk of myocarditis than the first dose, and (iii) men were at higher risk of post-vaccine myocarditis than women. In contrast to a recent study out of England [28], the adenovirus vector-based vaccines, Oxford-AstraZeneca and Janssen, did not impact the risk of either myocarditis or pericarditis.

### Strengths and limitations

In contrast with prior reports, this population-based study had the advantage of using prospectively collected information on vaccination and hospital admissions for an entire population, virtually eliminating recall and selection bias. Moreover, our study design compared periods of exposure and no exposure within the same subject, thus eliminating the variability between subjects that may arise when a group of exposed is compared to a control group of unexposed. However, this approach did not allow to evaluated the post-acute COVID-19 incidence of myocarditis and pericarditis. Finally, the use of registry data ensured a systematic evaluation of exposures, covariates, and outcomes. However, valid information on electrocardiography or cardiac imaging could not be obtained, so outcome misclassification could not be excluded. Moreover, information on myocarditis and pericarditis treatments were not available.

A potential bias in this observational study is that the decision to become vaccinated is an active personal choice, potentially confounding the association between vaccination and clinical outcomes [27]. Citizens who choose vaccination may be more health-conscious, leading to a healthcare-seeking bias that could overestimate the risk of myocarditis or pericarditis after vaccination if people with mild disease are more likely to be identified among vaccinated individuals [27]. In addition, the inability to compare those who chose to receive the vaccine and those who chose not to be vaccinated, may overestimate vaccine effectiveness and reduce the NNT value. However, the secondary analysis which excluded individuals who did not receive vaccination during the observation period, did not result in substantially different findings. Confounding may also be due to time-invariant characteristics, like socioeconomic status, that may differ between vaccinated and unvaccinated people. However, the Danish study showed that estimates generated from a self-controlled case series (SCCS) design, which took time-invariant characteristics into account, did not differ from those obtained from the usual cohort design, suggesting that unmeasured covariates were unlikely to impact the estimates. The secondary analysis conducted by the current study allowed for a comparison between exposure and no exposure periods within the vaccinated cohorts, avoiding the inclusion of unvaccinated individuals. The latter approach has some elements in common with the SCCS design but includes individuals who both experienced and did not experience the outcome, and observations were censored when the outcome occurred. This avoided the introduction of any biases resulting from the modification of the vaccine propensity due to the onset of myocarditis or pericarditis.

## Conclusions

Myocarditis and pericarditis rates were higher during periods of vaccine exposure (i.e. 28 days after receipt of an mRNA vaccine) than those observed during periods of no exposure. Increased myocarditis rates were particularly high for men 16 to 19 years of age within a few days after receiving the Moderna vaccine, particularly after the second dose. The balance between harms (myocarditis or pericarditis resolving in few days of hospital stay without any fatal outcome) and benefits (the prevention of severe acute respiratory syndrome which may result in invasive intubation or death) was almost always in favour of vaccination.

## Supplementary Information

**Additional file 1: Table S1**. Codes used for identifying cardiovascular comorbidities.

## Data Availability

The data that support the findings of this study are available from Lombardy Region, but restrictions apply to the availability of these data, which were used under license for the current study, and so are not publicly available. Data are however available from the Lombardy Region upon reasonable request.
