# Supplementary material for: Increased risk of myocarditis and pericarditis and reduced likelihood of severe clinical outcomes associated with COVID-19 vaccination: a cohort study in Lombardy, Italy
Source: BMC Infect Dis. 2022 Nov 12;22:844. doi: 10.1186/s12879-022-07823-3 (PMC9652976; doi:10.1186/s12879-022-07823-3)
Supplement: Supplementary file 1 — Additional file 1: Table S1. Codes used for identifying cardiovascular comorbidities. [file 12879_2022_7823_MOESM1_ESM.docx]

**Additional file 1: Table S1**. Codes used for identifying cardiovascular comorbidities

| **Disease/condition** | **ICD-9 CM code** | **DRG code** | **ATC code** | **Outpatient services code** | **Exemption code** |
| --- | --- | --- | --- | --- | --- |
| Arterial vascular disease | 440, 441, 442, 443.1, 443.2, 444, 445, 447  39.24, 39.25, 39.26, 39.50, 39.51, 39.52, 39.54, 39.56, 39.57, 39.58, 39.71, 39.72, 39.73, 39.74, 39.79, 39.90 (procedure) |  |  |  | 002.440, 002.441.2, 002.441.4, 002.441.7, 002.441.9, 002.442, 002.444, 002.447.0, 002.447.1, 002.447.6 |
| Heart failure | 428 |  | C09A, C09C (DDD>50%) and  [C03CA, C03CB, C03EB (DDD>50%) or  C07AG02 C07AB02 C07AB07 (DDD>50%)] |  | 021.428 |
| Familial and non-familial hypercholesterolaemia | 272.2, 272.4 |  | C10 |  |  |
| Valvular heart disease | 394-397, 745-747, V42.2, V43.3  35 (procedure) |  |  |  | 002.394, 002.395, 002.396, 002.397, 002.424, 002.745, 002.746, 002.747, 002.V42.2, 002.V43.3 |
| Venous vascular disease | 452, 453, 459.1 |  |  |  | 002.452, 002.453, 002.459.1 |
| Ischaemic heart disease | 410-414  36 (procedure) |  | C01DA (DDD>50%) |  | 002.414 |
| Arrhythmic myocardiopathy | 426, 427, V45.0  37.65, 37.66, 37.68, 37.70, 37.87, 37.89, 37.96 (procedure) |  | C01B (DDD>50%) | 89.48.1 | 002.426, 002.427, 002.V45.0 |
| Cardiomyopathy (not arrhythmia-induced) | 402, 404, 415-417, 425, 429.4 |  | C02KX01, C02KX02, C02KX03, G04BE03, G04BE08, B01AC09 (DDD>50%) |  | 031.402, 031.403, 002.416, 002.417, 002.429.4, 0031.402, 0031.403 |
| Hypertension | 401, 403, 405 | 134 | C02AC01, C02CA04, C03, C07, C08C, C09 (DDD > 50%) |  | 031.401, 031.405, D31.401, D31.405, 0031, 0031.405.0 |

# ICD-9 CM: International Classification of Diseases, Ninth Revision, Clinical Modification; DRG: Diagnosis-Related Group; ATC: Anatomical Therapeutic Chemical classification system; DDD: Defined Daily Dose; EX: exemption code
